# Improvement of erectile dysfunction by the active pepide from *Urechis unicinctus* by high temperature/pressure and ultra - wave assisted lysis in Streptozotocin Induced Diabetic Rats

**DOI:** 10.1590/S1677-5538.IBJU.2015.0606

**Published:** 2016

**Authors:** Kang Sup Kim, Woong Jin Bae, Su Jin Kim, Kyong-Hwa Kang, Se-Kwon Kim, Hyuk Jin Cho, Sung-Hoo Hong, Ji Youl Lee, Sae Woong Kim

**Affiliations:** 1 Department of Urology, College of Medicine, The Catholic University of Korea, Seoul, Korea; 2 Catholic Integrative Medicine Research Institute, The Catholic University of Korea, Seoul, Korea; 3 Marine Bioprocess Research Center, Pukyong National University, Busan, Korea; 4 Department of Marine-Bio, Convergence Science and Marine Bioprocess Research Center, Pukyong National University, Busan, Korea

**Keywords:** Erectile Dysfunction, Streptozocin, UFEII protein, Urechis unicinctus [Supplementary Concept]

## Abstract

**Introduction:**

We investigate the effect of active peptide from Urechis unicinctus (UU) by high temperature/pressure and ultra-wave assisted lysis on erectile dysfunction in streptozotocin-induced diabetic rats.

**Materials and Methods:**

Forty 12-week-old Sprague-Dawley rats were used in this study. Diabetes was induced by a one-time intraperitoneal injection of streptozotocin (50mg/kg). One week later, the diabetic rats were randomly divided into four groups: normal control, untreated diabetes control, and groups treated with 100 or 500mg/kg/d UU peptide. Rats were fed with UU peptide by intragastric administration for 8 weeks. After 8 weeks, penile hemodynamic function was evaluated in all groups by measuring the intracavernosal pressure after electrostimulating the cavernous nerve. Nitric oxide (NO) and cyclic guanosine monophosphate (cGMP) activities were measured and endothelial nitric oxide synthase (eNOS) and neuronal NOS (nNOS) protein expression was determined by Western blot.

**Results:**

Maximum intracavernosal pressure in diabetic control rats decreased significantly compared to normal control rats, and was increased significantly compared to untreated diabetic rats after UU peptide supplementation. Treatment with the higher dose of UU peptide significantly increased the NO and cGMP levels compared with the diabetic control group. Decreased activity and expression eNOS and nNOS were found in the diabetic rats compared with the normal control group. Decreased eNOS and nNOS in diabetic rats were improved by UU peptide administration.

**Conclusions:**

Active peptide from UU ameliorates erectile function in a streptozotocin induced diabetic rat model of erectile dysfunction.

## INTRODUCTION

Erectile dysfunction (ED) is defined as the consistent or recurrent inability to attain and/or maintain a penile erection sufficient for satisfactory sexual performance (1). About 52% of men between the age of 40 and 70 years suffer from ED. ED is closely associated with an increasing number of systemic diseases including cardiovascular disease, hypertension, diabetes mellitus (DM), hypercholesterolemia, and depression, as well as behavioral disorders including alcoholism, smoking, and drug abuse (2, 3).

DM is a metabolic disease characterized by hyperglycemic condition resulting from damages in insulin secretion and/or insulin resistance, and disturbance of carbohydrate metabolism. DM is a major risk factor of ED and the prevalence of ED in men with DM is significantly higher than in those without DM (4). The pathophysiologic mechanism of ED induced by DM may result from neurovascular dysfunction (5, 6). Endothelial nitric oxide (NO) synthase (eNOS) produces a physiological level of NO in endothelial cells under the normal circumstance. Rats in whom DM has been induced by chemical diabetogenesis show significant reductions in penile eNOS and neuronal NO synthase (nNOS), followed by markedly decreased NO production (5). Dysfunction occurring in penile endothelial cells induces ED. Therefore, DM has the potential to have an impact on all the components of ED.

Multiple treatment strategies have been established to treat ED induced by DM. These include DM control, psychosexual counseling, and medication with inhibitors of type-5 phosphodiesterase (PDE5) (7). Although therapy with PDE5 is effective for ED, treatment efficacy in patients with DM is significantly lower (8). Therefore, new therapeutic strategies are needed for patients with ED and DM.

**Figure 1:**
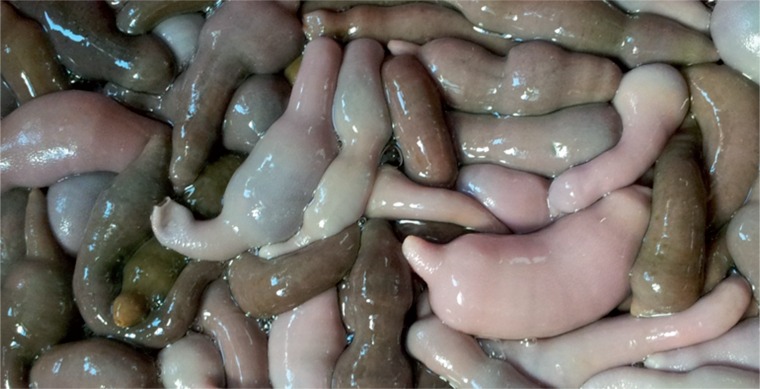
The picture of Urechis unicinctus.

Urechis unicinctus (UU) (Figure-1) is a member of the Echiuroidea, Xenopneusta, Urechidae and cyclindrical or oval shaped marine inverterbrates. They inhabit marine intertidal and subtidal zones of North China, Korea and Japan. UU contains amino acids, such as glycine, glutamic acid, and aspartic acid, and a component that is a new potential source of fibrinolytic agents, which has antithrombotic effects in an animal model of thrombosis (9). Glycosaminoglycan from UU has a hypoglycemic effect in diabetic mice (10) and an isolated peptide from UU may enhance erectile function in vitro by increasing the levels of NO and cyclic guanosine monophosphate (cGMP) (11). UU is considered a natural tonic in Asian countries and is considered to have positive effects on erectile function. However, detailed studies have not been conducted to explore the active mechanisms of UU peptide in vivo.

The purpose of this study was to determine whether UU peptide improves ED induced by DM via the NO/cGMP pathway and recovers the function of endothelium in the corpus cavernosum. The effect of UU peptide on corpus cavernosal smooth muscle tissues in rat with ED associated DM also was investigated.

## MATERIALS AND METHODS

### Preparation of Urechis unicinctus peptide

UU was generously obtained from a local aquatic market (Jagalchi) in Busan, Republic of Korea. UU was washed and homogenated with the addition of water. Salt water was removed and UU was cut into 0.8-l. 0cm sized portions, which were pressurized and autoclaved at a pressure of 1kgf/cm^2^ at a temperature of 121°C for 20 minutes. The preparation was ultrasonicated at a frequency of 40KHz for 60 minutes, vacuum filtered and freeze-dried. The resulting UU peptide was used for the experiments.

### Animals experiments and diet

Forty 12-week-old adult male Sprague-Dawley rats weighing 250-300g were obtained from Orientbio Inc. (Seongnam, Korea). The study was designed and conducted in accordance with the Public Health Service Policy on Human Care and Use of Laboratory Animals (NIH Office of Laboratory Animal Welfare, revised 2002). Rats were handled according to the rules and regulations of the Institutional Animal Care and Use Committee in School of Medicine, The Catholic University of Korea. Animals were housed two per cage and maintained on a 12-h light-dark cycle with ad libitum access to water and food. After one week acclimatization, the rats were randomly divided into four groups of four rats each: untreated (normal control), streptozotocin (STZ) induced diabetes control (DM control), STZ induced diabetic rats administered with 100mg/kg of UU peptide (UU 100) and STZ induced diabetic rats administered with 500mg/kg of UU peptide (UU 500). All rats in the DM and DM+UU groups received a single intraperitoneal injection of STZ (50mg/kg). Glucose in blood drawn from the tail vein and body weight was checked weekly and significant diabetes (serum glucose >250mg/dL) was confirmed in all rats within 1 week. This diabetic serum glucose level was maintained throughout the experiment period. One week after the induction of diabetes, rats in the UU group were treated with daily oral UU peptide (100mg/kg or 500mg/kg) dissolved in 10mL distilled water and administered orally through an 8F oral zonde needle once a day for 8 weeks. After 8 weeks, intracavernosal pressure (ICP) and mean arterial pressure (MAP) measurements were performed in all 40 rats. After the measurements were completed, the penis of each rat was excised at the level of the crus and blood samples were collected. The excised penis was cut and divided into two portions. One portion was used for measurement of NO/cGMP. The other portion was used for histologic examination and Western blotting. The separated penile tissues were stored in-70°C liquid nitrogen until needed.

### Assessment of ICP and MAP

Erectile function was evaluated by tracing the ICP under cavernous nerve electrical stimulation under anesthesia at 8 weeks after inducing DM and administrating UU peptide. Rats were anesthetized with Tiletamine (50mg/kg, Zoletil^®^; Virbac, Carros, France) intraperitoneally and anesthesia was retained with supplemental Tiletamine as needed. The major pelvic ganglion and cavernous nerves lateral to the right prostate were revealed and identified using a lower abdominal midline incision with the rat in the supine position. The shaft of the penis was removed of skin and fascia, and the corpus cavernosum and crus of the penis were exposed. The left corpus cavernosum of the proximal portion of the penis was cannulated by insertion of a 250IU/mL heparinized 23-gauge butterfly needle connected to a pressure transducer to assess ICP. The right carotid artery via a midline neck incision was cannulated with a PE-50 catheter filled with 250IU/mL heparinized saline to measure MAP. A hook-shaped bipolar silver electrical stimulator was placed on the pelvic ganglion to stimulate the cavernous nerve at 10V for 50s and 2.4mA with a pulse width of 0.5ms. Cavernous nerve stimulation was performed at least three times and the interval between stimulations was maintained for over 10 minutes. The peak ICP during nerve electrostimulation was calculated by an isometric force transducer and recorded on a computer using a Power Lab^®^ data acquisition system (AD Instruments Pty Ltd., Oxford, UK). ICP and MAP ratios were analyzed using Chart 5 software (AD Instruments Pty Ltd.). After completion of functional analysis, the corpus cavernosum was removed and divided into two portions.

### NO measurement

Blood samples obtained from the inferior vena cava were centrifuged at 3.000rpm for 10-15 minutes to separate the serum. An equivalent volume of methanol was added to the plasma and mixed well to remove serum proteins, and centrifuged at 15.000rpm for 10-15 minutes. The concentration of NO in the supernatant was measured with a model ENO-20NO analyzer using high-performance liquid chromatography (Eicom, Kyoto, Japan).

### Measurement of cGMP in the corpus cavernosum

After assessment of ICP and MAP, corpus cavernosum tissue was immediately removed and frozen at-70°C in liquid nitrogen. A commercial cGMP direct immunoassay kit (K372-100; BioVision, Milpitas, CA, USA) was used to measure the cGMP level as detailed by the manufacturer.

### Determination of eNOS and nNOS protein expression by Western blot analysis

The corpus cavernosum was collected from all rats and homogenized individually in a buffer solution of 32mM sucrose, 20mM HEPES, (pH 7.4), 1mM EDTA, 1mM dithiothreitol, 10μg/mL leupeptin, 2μg/mL aprotinin, 10μg/mL trypsin inhibitor, 1μg/mL pepstatin and 1mM phenylmethylsulfonyl fluoride. The homogenized buffer solution was stored on ice for 15 min and centrifuged at 4°C for 13.000rpm for 15 minutes. The supernatant solution was dissociated and amount determined to contain 30μg protein was boiled at 95°C for 5 minutes and proteins resolved by 12% discontinuous sodium dodecyl sulfate polyacrylamide electrophoresis (SDS-PAGE). The proteins were transferred into a 0.2μm polyvinylidene fluoride membrane (Amersham Bioscience, Piscataway, NJ, USA) for 150 minutes at 25V. Each membrane was reacted with blocking buffer (5% skim milk in Tris buffered saline-Tween 20, TBST) for 30 minutes at room temperature. eNOS or nNOS (BD Biosciences, San Jose, CA, USA) antibody, or beta-actin (1:10000; Santa Cruz Biotechnology, Santa Cruz, CA, USA) were added for 2 hours and the membrane was washed three times using full term for TBST at intervals of 10 min. The secondary antibody, either anti-mouse IgG or anti-goat IgG conjugated to horseradish peroxidase (1:2000; Zymed Laboratories, South San Francisco, CA, USA) were added at room temperature for 1 hour. Each membrane was washed six times using TBST with an interval of 5 minutes between each washing. Enhanced chemiluminescence was conducted using ECL Western blotting detection reagents. Densitometric analysis of band intensity was carried out using the Luminescent Image Analysis System (LAS-3000; FUJI Film, Tokyo, Japan) equipped with Multi Gauge 3.0 software. The intensity of the bands was measured and the expression of proteins including the differences between the control and experimental groups were analyzed.

### Masson's trichrome staining of corpus cavernosum

The skin-free middle portions of the penile shafts were fixed overnight in 10% formalin and washed and stored in 70% alcohol at 40°C until processed for paraffin-embedded tissue sectioning. After the fixed cavernosal tissue was embedded in paraffin wax, 5-μm cross-sections of the cavernosal tissue was obtained for Masson trichrome staining to evaluate muscle fibrosis. For quantitative image analysis, stained sections were photographed using a model BX50 microscope (Olympus, Tokyo, Japan). Saved images were analyzed using Photoshop CS6 (Adobe Systems Incorporated, San Jose, CA, USA). Red and blue pixel numbers of the corpus cavernosum were distinguished and analyzed for smooth muscle (stained in red) and collagen (stained in blue) and the cavernous smooth muscle-to-collagen fiber ratio was calculated using Panoramic viewer 1.14 software (3DHISTECH Ltd., Budapest, Hungary). The ratio was evaluated three to four times for each specimen, and is presented as the mean±standard deviation (SD).

#### Statistical analysis

All data are presented as the mean±SD. For comparison of the four groups, analysis of variance test was used. If the results of the analysis of variance test were significant, Bonferroni multiple comparison's test was used. All calculations were performed using SPSS statistical software (SPSS, Chicago, IL, USA). Differences of P <0.05 were considered statistically significant.

## RESULTS

### Measurements of body weight and blood glucose level

Body weight and blood glucose level of the rats at the beginning of administration of UU peptide and during the course of administration are shown in Table-1. At 8 weeks, body weight increase in all DM groups was significantly lower than that of the control group (P <0.05). Furthermore, all DM+UU groups had significant higher weight gain than that of the DM group (P <0.05). Eight weeks after UU peptide administration, the blood glucose levels in DM+UU 500 group were significantly lower than in that of the DM group (P <0.05). The increasing blood glucose level in all DM+UU groups were significantly lower than in that of the DM group at 8 weeks of the experiment.

**Table 1:** Changes in Body Weight and Serum Glucose Levels in the Experimental Groups

		Pre-DM	4 weeks after DM	8 weeks after DM
**Body weight (g)**				
	Control (n = 10)	314 ± 8	370 ± 9	552 ± 20
	DM control (n = 10)	328 ± 11	353 ± 20	424 ± 20
	DM+UU100 (n = 10)	314 ± 5	351 ± 22	471 ± 14* ^,^ #
	DM+UU 500 (n = 10)	318 ± 9	349 ± 20	477 ± 13* ^,^ #
Serum glucose (mg/dL)				
	Control (n = 10)	123.8 ± 3.3	120.8 ± 1.5	121.3 ± 2.9
	DM control (n = 10)	120.8 ± 11.8	395 ± 8	497.5 ± 12.1
	DM+UU100 (n = 10)	126.5 ± 1.3	381 ± 16.4*	457 ± 21.9* ^,^ #
	DM+UU 500 (n = 10)	124.1 ± 6.2	324 ± 1.2* ^,^ #	386.4 ± 159.8* ^,^ #

DM, diabetes group; DM + UU, diabetes group treated with UU.

* Significant difference (P < 0.05) compared with the control group.

# Significant difference (P <0.05) compared with the DM group.

### Erectile function assessment

**Figure 2:**
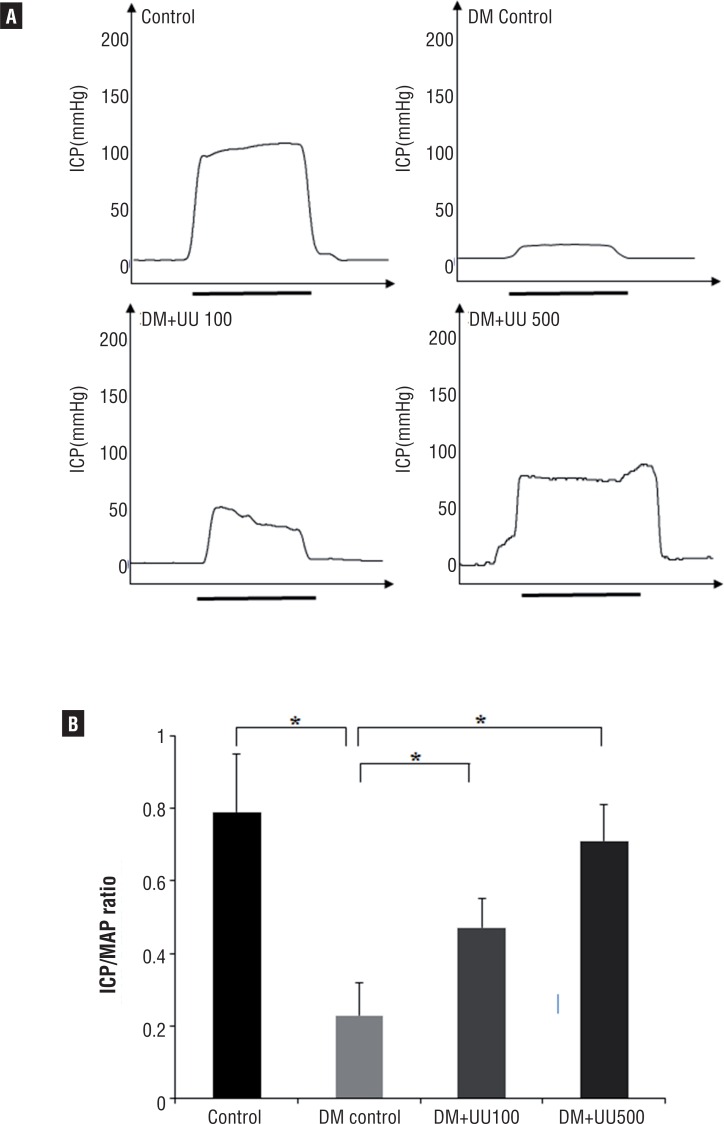
(A) Measurement of ICp under cavernous nerve stimulation at 10v after 8 weeks administration of UU peptide. The black line represents electrical stimulation interval of 50s. (B) The ratio of ICP/MAP during electrical stimulation were calculated for each group *p <0.05.

To assess erectile function, ICP and ICP/MAP ratio in response to cavernous nerve electrostimulation were measured after 8 weeks administration of UU peptide. Figure-2 graphically depicts the ICP/MAP ratios. No significant differences in MAP were observed between the groups. The ICP/MAP ratio in the control group (0.79±0.16) was significantly higher compared with the DM and DM+UU100 groups (P <0.05). The DM control group had a significantly decreased ICP/MAP ratio (0.23±0.09) compared with the other groups. The ICP/MAP ratio of the DM+UU 100 (0.47±0.08) and DM+UU 500 (0.71±0.1) group was both significantly higher compared with the DM group (P <0.05). Moreover, there were no statistically significant differences in the ICP/MAP ratio between the control group and the DM+UU 500 group (P=0.453).

### NO level

**Figure 3:**
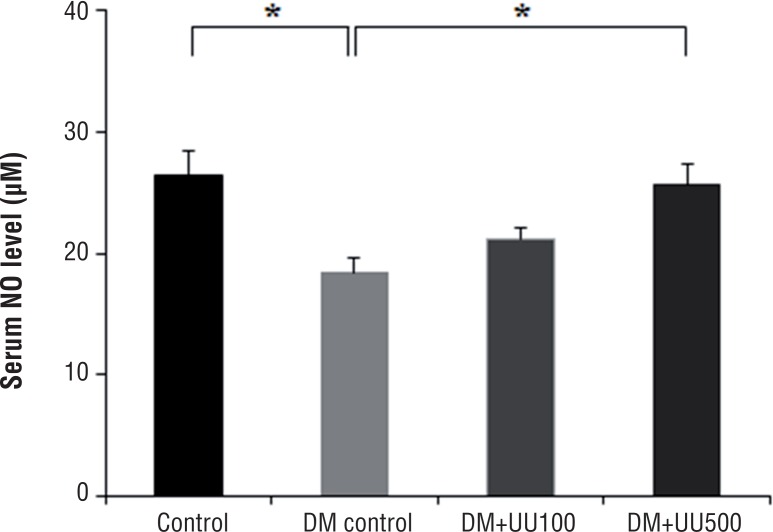
Serum concentration of NO at 8 weeks after administration of UU peptide *P <0.05.

NO concentrations in serum are summarized in Figure-3. Compared with the control group, the DM group had significantly decreased NO level (26.4±2.1 versus 18.4±1.2, P <0.05). At the same time, the DM+UU500 group had significantly increased NO level compared with the DM control group (25.1±1.6 versus 18.4±1.2, P <0.05). However, DM+UU100 group did not show significant difference of NO compared with the DM group. Furthermore, there is no significant difference between control group and DM+UU500 group (P=0.573).

### Level of cGMP in the corpus cavernosum

**Figure 4:**
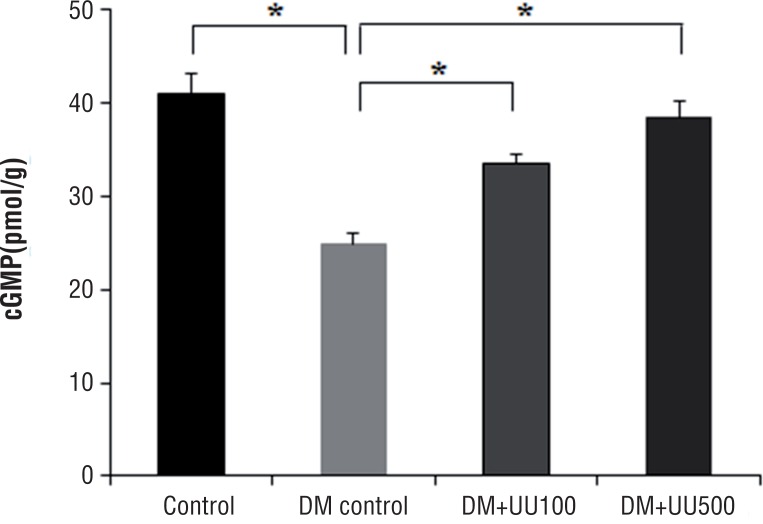
Cyclic guanosine monophosphate (cGMP) concentration in the corpus cavernosum at 8 weeks after administration of UU peptide *P <0.05.

The corpus cavernosum concentration of cGMP (pmoL/g) in the control, DM control, DM+UU100 and DM+UU500 was 41.0±2.1, 24.9±1.2, 33.5±1.0 and 38.5±1.6, respectively (Figure-4). The concentration of cGMP in the corpus cavernosum was significantly increased in the DM+UU500 group compared with DM control group (P <0.05). However, there was no significant difference between the control and DM+UU500 group (P=0.421).

### eNOS and nNOS protein expression

**Figure 5:**
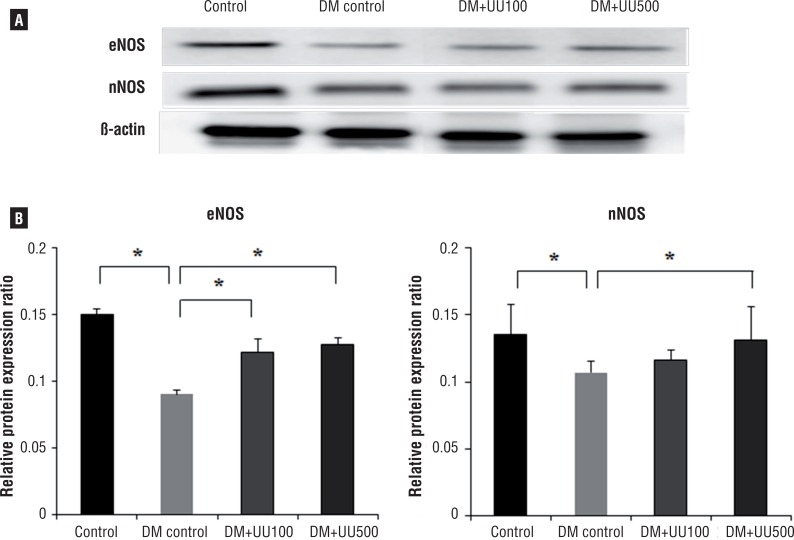
Western blot analysis of eNOs and nNOs in corpus carvernosum at 8 weeks-control, DM control, UU+100, UU+500 (A), Densinometric analysis to β-actin of eNOs and nNOs (b) *P <0.05.

Densitometric analysis quantified the expression of eNOS and nNOS (Figure-5). Decreased expressions of eNOS and nNOS were observed in the DM control group compared to the control group (P <0.05). Increased expression of the eNOS and nNOS were evident in the DM+UU100 and DM+UU500 groups compared with DM control group (P <0.05). However, the densitometric results of rats administered with 100mg did not reach the level of the control group.

### Smooth muscle – to - collagen ratio in the corpus cavernosum tissue

**Figure 6:**
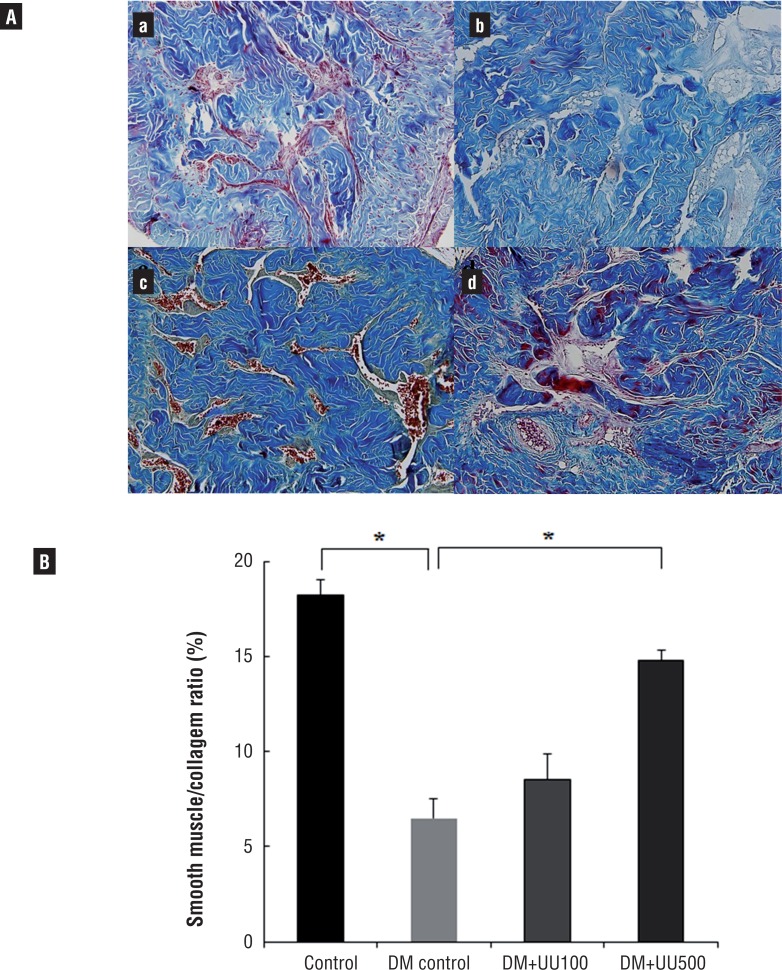
(A) The Masson's trichrome staining of corpus cavernosum in control (a), DM control (b), DM+UU100 (c) and DM+UU500 (d) groups after 8 weeks. smooth muscle was stained red color and collagen fibers were stained purple-blue color. (B) Quantitative analysis results of smooth muscle/collagen ratio in each group *P <0.05.

The content of collagen tissue and smooth muscle in the penile corpus cavernosum tissue was observed by Masson's trichrome staining (Figure-6). In the DM control group, cavernous tissue revealed a much higher density of collagen tissue compared with the control group. These results suggested that DM induces the deposition of collagen in corpus cavernosum with smooth muscle atrophy. The smooth muscle-to-collagen ratio in the DM+UU500 group was higher than in the DM group (14.8±0.5 versus 6.5±1.0, P <0.05). However, there was no significant difference in the ratios between the DM control group and DM+UU100 group. Additionally, the ratio in the DM+UU500 group was lower than in the control group (P <0.05).

## DISCUSSION

The main findings of this present study demonstrate that the oral administration of UU peptide by high temperature/pressure and ultra-wave assisted lysis improves erectile responses in STZ-induced diabetic rats through the elevation of the NO/cGMP signaling activity and increased expression and activity of cavernosal eNOS and nNOS in penile tissues. Improvement of erectile function was also demonstrated by functional and histological approaches after oral administration of UU peptide. To our knowledge, this is the first demonstration that oral administration of UU peptide can enhance erectile ability in vivo.

Normal penile erection is a hemodynamic event that is dependent on penile smooth muscle relaxation mediated by NO/cGMP signaling, parasympathetic neurotransmission and other regulatory factors (12). Corpus cavernosum smooth muscle relaxation begins and is retained by an increased NO production through the activation of eNOS and nNOS by sexual stimulation (13, 14). NO stimulates soluble guanylate cyclase, subsequently increasing cGMP levels in smooth muscle (15). Relaxation of smooth muscle, increased penile arterial blood flow and restricted venous outflow generate penile erection. Dysfunction of the NO/cGMP pathway is widely regarded as one of the causes of erectile dysfunction (16). DM may lead to ED by a number of pathophysiological events including neuropathy, endothelial dysfunction, hormonal change and cavernosum smooth muscle structural/functional changes (17, 18).

Although the exact mechanisms of endothelial dysfunction in the penis are not fully understood, hyperglycemia is recognized as the primary cause in the pathogenesis of the endothelial dysfunction of the vasculature in DM (19). Therefore, it is important to control hyperglycemia to prevent and treat ED associated with DM. Romeo et al. (20) reported that glycemic control is an independent factor in the ED; a severe glycemic control group displayed a significant lower incidence of ED compared with a conventional treatment group. However, retaining a slightly higher glucose level than the normal glucose level is recommended according to the guidelines of the DM treatment (21, 22). Glycemic control with an additional therapy is a more ideal therapeutic strategy for the management of ED associated DM. Presently, UU showed a significant hypoglycemic effect in STZ-induced hyperglycemic rats. This result may be caused by glycosaminoglycan, which possesses hypoglycemic activity associated with improved antioxidant ability of DM mice, increasing superoxide dismutase activity and decreasing malondialdehyde, recovering liver and pancreatic tissue, enhancing capacity of the glucose tolerance and insulin sensitivity (10). In our study, we also confirmed that serum glucose level in the DM+UU groups were significantly lower than in that of the DM group (Table-1). Although serum glucose level in diabetic rats receiving UU peptide groups was lower than in that of the DM group, the higher glucose level compared with normal group should be managed by another treatment modality. In addition, Cho et al. (23) reported that well-controlled glucose level of diabetic rats affects recovery from ED associated with DM, and that strict glucose control can influence recovery from ED to almost normal status. Therefore, oral administration of UU peptide added to standard glycemic control treatment may be beneficial for treating ED associated with DM patients.

In the current study, ICP/MAP ratios were checked in response to electrostimulation to examine erectile function. ICP and ICP/MAP ratios were significantly increased in the DM+UU groups, suggesting that UU peptide likely recovers the erectile response in the ED associated DM rats. Furthermore, 500mg UU peptide administration represented more effective than 100mg UU peptide administration in the recovery of ICP.

In a recent in vitro study of UU peptide related to the erectile function (11), high temperature (125.86°C) and pressure (1.13kgf/cm^2^) UU lysate treated endothelial cells displayed up to 1.5-fold increased NO activity. Furthermore, smooth muscle cells treated with UU lysis after ultrasonic assisted breakdown of high temperature/pressure displayed increased cGMP activity and decreased Ca^2+^activity. To confirm the bioactive peptide related the erectile function properties from UU lysate, chromatographic methods were used and the amino acid sequence associated with potent activity confirmed. In vitro, amino acid treated cells displayed a dose-dependent increase in NO and cGMP, and dose-dependent decrease in activities associated with Ca^2+^. The authors concluded that acquired active peptide from high temperature/pressure and ultrasonication assisted UU peptide improves erectile function as a response to the observed increased and decreased activities. With this in vitro background, we prepared UU peptide to confirm the improvement of erectile dysfunction in vivo. Oral UU peptide supplementation increased the NO levels in the serum in a dose-dependent manner (Figure-3). Furthermore, cGMP levels in the corpus cavernosum revealed similar results (Figure-4). It has been reported that systemic NO level correlated with NO levels in the corpus cavernosum (24). Therefore, the positive effect of erectile function by oral UU peptide supplementation may be due to relaxation mediated by NO and cGMP production in the corpus cavernosum.

The different isoforms and subtypes of NOS include neuronal (nNOS), inducible (iNOS) and endothelial (eNOS) (25, 26). NO release in the penile vascular and non-adrenergic non-cholinergic system is regulated by eNOS and nNOS (25). eNOS is mainly found in the endothelium of the vessels of the penis and is also present in cavernosum smooth muscle (3, 27). nNOS has been proposed to be important, compared with the other NOS isoforms, in promoting relaxation of the corpus cavernosum and inducing increased blood flow, which facilitates penile erection (28). eNOS and nNOS mediated cavernosum smooth muscle relaxation is impaired in a rat model of diabetes (29). Presently, diminished penile expression and activity of eNOS and nNOS in corpus cavernosum tissues were markedly increased after administration of UU peptide to diabetic ED rats, with NO production being increased (Figure-5). The mechanism of positive effects is unclear, but is likely due to the function of the another specific peptide from UU. These findings might mean that UU can modulate the activity or the expression of enzymes like nNOS and eNOS that are abundant in penile tissues.

We examined penile cavernosal structure by Masson's trichrome staining. Smooth muscle of the corpus cavernosum plays an important role in the erection and relaxation of the penis. Fibrosis and loss of corpus cavernosum smooth muscle have been observed in patients with ED and the percentage of corpus cavernosum smooth muscle declines with age (30, 31). The mean weight of cavernosal tissue strips harvested from diabetic rats is reportedly less than that from control rats (6). This finding is associated with the loss of corporal SM. Presently, the DM control group showed a decreased smooth muscle-to-collagen ratio compared with normal control group. Oral UU peptide supplementation restored the ratio in the ED induced by DM rats. Since UU peptide increasing NO production (11) and NO enhances angiogenesis and promotes release of other angiogenic growth factors (32), the increased NO level after oral UU peptide supplementation might lead to the development of angiogenesis in corpus cavernosum tissue. This might be the mechanism of the improved smooth muscle-to-collagen ratio of the corpus cavernosum.

The main limitation of this study is the focus on the effect of UU peptide in endothelial dysfunction. Other mechanisms involved in ED associated with DM, such as diabetic neuropathy, were not examined. Finally, the STZ-induced diabetic rat model mimics type 1DM. Further studies should investigate the role of UU peptide administration in type 2DM, which is the predominant form of DM.

Our results suggest that UU peptide leads to a significant reduction in serum glucose levels in diabetic rats and the mechanism underlying the therapeutic effects of UU peptide involves the NO/cGMP signaling pathways. UU peptide may be potentially valuable in the natural rejuvenation of diminished erectile function. While future clinical trials are needed to determine the efficacy and safety of UU peptide for clinical use, our results suggest that UU peptide is a viable adjuvant therapeutic option to current treatment for ED.

## CONCLUSIONS

The study is the first to suggest that the peptide extracted from Urechis unicinctus, may have a potency to improve the erectile function in streptozotocin-induced diabetic rat erectile dysfunction model. Treatment with UU peptide improves maximal intracavernosal pressure by increasing NO and cGMP activity. Furthermore, treatment with UU peptide benefited eNOS and nNOS expression and smooth muscle distribution in the corpus cavernosum. Further studies are necessary to identify the active component in the UU peptide for ED.

## Abbreviations

ED: = erectile dysfunction
DM: = diabetes mellitus
NO: = nitric oxide
eNOS: = endothelial nitric oxide synthase
nNOS: = neuronal nitric oxide synthase
PDE5: = type-5 phosphodiesterase
UU: = Urechis unicinctus
cGMP: = cyclic guanosine monophosphate
ICP: = intravavernosal pressure
MAP: = mean arterial pressure
SD: = standard deviation
